# Caesarian section (CS) delivery in Bangladesh: A nationally representative cross-sectional study

**DOI:** 10.1371/journal.pone.0254777

**Published:** 2021-07-15

**Authors:** Foyez Ahmmed, Muhammad Mahabub Rahaman Manik, Md. Jamal Hossain

**Affiliations:** 1 Department of Statistics, Comilla University, Kotbari, Cumilla, Bangladesh; 2 Department of Pharmaceutical Chemistry, Faculty of Pharmacy, University of Dhaka, Dhaka, Bangladesh; Queensland University of Technology, AUSTRALIA

## Abstract

A growing trend in the caesarian section (CS) for delivery is a threat to child health as well as maternal health. This study was conducted to identify the potential socioeconomic and demographic factors associated with CS delivery in Bangladesh. Data obtained from the Bangladesh Demographic and Health Survey (BDHS) 2014 has been used for this study. The prevalence of CS delivery among Bangladeshi mothers was 24% (Urban: 36.9%, Rural: 17.9%). A two-level logistic regression showed that mothers having delivery in the private sector or private hospital (adjusted odds ratio [AOR] = 38.70, 95% confidence interval [CI] = 29.58 to 50.62), mother’s age 25–35 years (AOR = 1.73, 95% CI = 1.26 to 2.37), wealth index average (AOR = 1.61, 95% CI = 1.15 to 2.27) and rich (AOR = 1.80, 95% CI = 1.29 to 2.51), antenatal visit 1–2 (AOR = 2.31, 95% CI = 1.47 to 3.64) and ≥ 3 (AOR = 3.68, 95% CI = 2.35 to 5.76), overweight mothers (AOR = 1.44, 95% CI = 1.09 to 1.90), multiple births (AOR = 3.87, 95% CI = 1.15 to 12.58), husband’s occupation professional/technical/managerial (AOR = 1.68, 95% CI = 1.15 to 2.47) were significantly more prone to CS delivery. Also, place of residence, number of family members, birth order, child’s size during birth, and divisions of Bangladesh, were potentially associated with CS delivery. The current epidemiological findings and evidence suggest adopting and implementing some urgent clinical practices and strict guidelines in the healthcare system to avoid unnecessary CS delivery in Bangladesh.

## Introduction

Although CS as a mode of delivery is an essential surgical process to reduce the risks associated with childbirth, an increasing trend in CS delivery has continued to boost worries worldwide. CS delivery is a life-saving intervention as a mode of delivery to reduce maternal and newborn death. Still, in the same breath, unnecessary CS delivery is threatening for both mother and child’s wellbeing [1]. Failing to conduct CS delivery on time may result in perinatal asphyxia, stillbirth, uterine rupture, or obstetric fistula, a marker for exceptionally prolonged and obstructed labor [2]. According to the World health organization (WHO), when medically necessary, CS must be conducted without striving for any specific rate for the mother in need [3]. However, CS delivery has some short-term and long-term risks, particularly in countries that lack adequate health facilities. Studies show that CS delivery rate, at the population level, exceeding 10% is not associated with the reduction in maternal and neonatal mortality rates [4, 5]. Rather rates of preterm delivery and neonatal mortality both rise with the increase of CS delivery [1, 6, 7].

Recently in Bangladesh, it is noticeable some mothers go under CS delivery without any complications. Several studies conducted in Bangladesh have identified a number of socioeconomic and demographic factors associated with CS delivery, such as mothers’ education, place of residence, wealth index, mothers’ age, age at marriage, age at first birth, parity, birth order, antenatal visit, husband occupation, delivery in the private sector, religion, geographical region [8–12]. Also, some other studies available in the literature for several countries identified various socio-economic, demographic, physical facilities, and community interrelated factors significantly associated with making decisions to undergo institutional delivery [13–16].

Now the question is, when CS delivery justifiable? There are some well-defined maternal indications when the adoption of CS delivery is acceptable [17]. Another effective procedure is Robson’s classification system to reduce the CS delivery rate [18–20]. According to WHO, Robson’s classification is the most appropriate system to classify CS to meet international and local needs [21].

The sustainable development goal 3 (SDG-3) aims to reduce the neonatal mortality to below 12 per 1000 live births and under-5 mortality to below 25 per 1000 live births and reduce the global maternal mortality ratio to less than 70 per 100000 live births. In Bangladesh, neonatal mortality, under-5 mortality rates are 23 and 38 per 1000 live births, respectively, and maternal mortality is 173 per 100000 live births in 2017 [22, 23], which is far-reaching to achieve. So, the increasing trend in CS delivery could be an obstacle to achieve these goals.

The foremost objective of this study is to determine the prevalence of CS delivery based on different background characteristics and find the potential socioeconomic, demographic, institutional, and social-network-related factors associated with the high rate of CS delivery so that decision-makers can take proper steps regarding this issue.

## Materials and methods

### Data and variables

This study used Bangladesh Demographic and Health Survey (BDHS) 2014 data. Information on data will be available at https://dhsprogram.com/data/available-datasets.cfm. After receiving ethical approval from the National Institute of Population Research and Training (NIPORT) of the Ministry of Health and Family Welfare, Bangladesh, the survey was conducted. Permission was taken from administrative offices, and verbal consent from each participant of the study was obtained before collecting data. The survey used a two-stage stratified cluster random sampling. In the first stage, 600 Enumeration Areas (EAs)/clusters (393 from rural and 207 from urban areas) were randomly selected. In the second stage, 30 households from each of the selected clusters were randomly selected, and each of the ever-married women (age 15 to 49 years) in the selected households were interviewed. Details of the sampling frame, sampling design, EAs, households, and questionnaires are available in the publicly available reports of the mentioned survey. The survey finally collected information from 17886 ever-married women of 15–49 years old, of which 4493 women (having at least one birth in the last three years from 2014) have information on the mode of delivery. Some missing values and abnormal data points have been detected in the considered covariates thereafter eliminated, and a total of 4433 women with complete information have been considered for this current study.

Since the study aims to find out the socio-economic and demographic factors associated with CS delivery, our response variable is the mode of delivery which is a dichotomous variable “1” for CS delivery and “0” for vaginal delivery.

Apart from the variables that were indicated in the previous studies, some new variables have been considered in this study. Variable Division contains six geographical regions (Dhaka, Chittagong/ Chattogram, Barishal, Khulna, Rajshahi, Rangpur, Sylhet). Age of mothers was categorized into three categories: less or equal to 24 years, 25 to 35 years, and greater or equal to 36 years. Place of residence contains two categories: urban and rural areas. Variable wealth index was calculated by principal component analysis based on the assets possessed by the households and categorized as poor (1st and 2nd quintiles), average (3rd quintile), and rich (4th and 5th quintiles). The Body Mass Index (BMI) of the mothers categorized into three categories: Normal (18.5 kg/m^2^ ≤ BMI <25 kg/m^2^), Underweight (< 18.5 kg/m^2^), and Overweight (≥ 25 kg/m^2^). The size of child during birth (larger, average, smaller) was calculated from the mothers’ perceptions as BDHS-2014 didn’t calculate children’s weight at birth. In variable Delivery in the private sector or hospital, we considered two categories: yes and no. Variable exposure to media was coded as ‘yes’ if a respondent was exposed to at least one of the three media: reading newspaper or magazine, listening to the radio, and watching television; otherwise ‘no’. Wanted index child was categorized as yes and no. Variables mother’s education level (no education, primary, secondary, and higher), religions (Muslim, non-Muslim), number of family members (less than or equal to 5 members, 6–10 members, and greater than or equal to 11 members), birth orders (1st, 2nd, and 3rd or above), age at first child’s birth (less than 20 years, greater or equal to 20 years), antenatal care (ANC) visits (No, 1–2, 3 or more), age at first marriage (less than or equal to 18 years, greater than 18 years), husband’s education levels (no education, primary, secondary or higher), type of birth (single birth, multiple births), gender of child (male, female), mother’s working status (yes, no), husband’s occupation (service/skilled and unskilled manual, professional/technical/managerial, agricultural work, none/other) have also been considered in this study.

Moreover, we had a look at the timing of decision for CS delivery and categorized into four options: on the day of delivery, a day before delivery, 2 to 7 days before delivery, and 8 to 30 days before delivery. We also considered variables to see the decision-makers and the reasons behind their decisions.

### Statistical analysis

We have constructed frequency distribution (percentage) for obtaining the prevalence of CS delivery among Bangladeshi women of reproductive age. Chi-square (χ^2^) test has been carried out to find the association between response variables and covariates. A single-level model might not be appropriate for analyzing the BDHS-2014 data as the obtained data was collected in the survey in several hierarchies. Because in multi-stage sampling, respondents are usually nested with the higher-order clusters. Therefore, to consider the cluster effects, a two-level logistic regression has been implemented to evaluate the impact of socio-economic and demographic factors on CS delivery. Level-I was regarded as an individual level and level-II as EAs or clusters level. To check the clustering effects, we assessed the Median odds ratio (MOR), one of the widely used methods [24, 25]. The formula for calculating MOR is as following-

$$MOR=exp\left(\Phi^{-1}(0.75)\sqrt{2\sigma_{B}^{2}}\right)$$

Where Φ^−1^(0.75) = 0.6745, is the 75^th^ percentile of the standard normal distribution, and $\sigma_{B}^{2}$ is the cluster variance. MOR value is always greater or equal to one. MOR value one indicates that there is no cluster effect. MOR greater than one means the presence of clustering effects and needs to be removed to get more accurate results [24]. All the analyses have been done by using STATA statistical package (version 15) and SPSS (version IBM 20).

## Results

### Association between CS delivery and different factors

The association of the mode of childbirth of the married women (reproductive age: 15–49 years) in Bangladesh with different socioeconomic and demographic factors along with the descriptive statistics has been delineated in Table 1. The χ^2^ values and the corresponding *p*-values have also been included in the table to assess the significance of the association. A sample of 4433 individuals has been analyzed in this study. Among them, 1065 (24%) had CS delivery, and the remaining 3368 (76%) had a vaginal delivery. According to the geographical region, the proportion of CS delivery was the highest in Khulna (35.2%), followed by Dhaka (32.9%), Rajshahi (28%), Chittagong/Chattogram (20.1%), Barishal (19.90%), Rangpur (19.50%), and Sylhet (13.30%). The corresponding *p*-value (< 0.001) obtained from the χ^2^ test indicated a significant association between mode of delivery and geographical region. We have also found that mothers aged 25 to 35 years old had the highest rate (26.50%) of CS delivery, and mothers’ age was significantly (*p* < 0.01) associated with mode of delivery. Mothers who live in the urban area had a higher proportion of CS delivery (36.9%) compared to the mothers living in the rural area (17.9%), and this difference (19%) was also statistically significant (*p* < 0.001). Higher educated mothers were more likely to have CS as a mode of delivery (57.7%), and the education level was significantly associated with mode of delivery (*p* < 0.001). The scenario was also similar to husbands’ educational qualifications: the higher the study level, the higher rate of CS. Besides, religion was also significantly associated with mode of delivery (*p* < 0.05), and Muslim mothers had a lower rate of CS delivery compared to non-Muslim mothers (23.60% vs. 28.50; *p* < 0.05). Furthermore, mothers who live in a family having five or fewer members had a higher rate of CS childbirth (25.30%), where the number of family members has also significantly impacted the mode of delivery (*p* < 0.05).

**Table 1:** Association of the mode of delivery with socioeconomic and demographic factors for the ever-married women of reproductive age in Bangladesh (N = 4433).

Factors	Categories	Total (%)	CS count (%)	χ^2^ value	*p-*value
**Mode of Delivery**	Caesarian section	1065 (24)			
	Vaginal	3368 (76)			
**Divisions**	Dhaka	780 (17.60)	257 (32.90)	134.63	< 0.001
	Chittagong/Chattogram	849 (19.15)	171 (20.10)		
	Barishal	527 (11.89)	105 (19.90)		
	Khulna	528 (11.91)	186 (35.20)		
	Rajshahi	539 (12.16)	151 (28.00)		
	Rangpur	543 (12.25)	106 (19.50)		
	Sylhet	667 (15.05)	89 (13.30)		
**Mother’s Age (years)**	≤ 24	2433 (54.88)	545 (22.40)	9.71	0.008
	25–35	1792 (40.42)	474 (26.50)		
	≥ 36	208 (4.69)	46 (22.10)		
**Place of Residence**	Urban	1421 (32.06)	525 (36.90)	191.31	< 0.001
	Rural	3012 (67.94)	540 (17.90)		
**Maternal Education (level of schooling)**	No education	597 (13.47)	45 (7.50)	509.106	< 0.001
	Primary	1223 (27.59)	152 (12.40)		
	Secondary	2102 (47.42)	573 (27.30)		
	Higher	511 (11.53)	295 (57.70)		
**Religions**	Muslim	4075 (91.92)	963 (23.60)	4.258	0.039
	Non-Muslim	358 (8.08)	102 (28.50)		
**Number of Family Members**	≤ 5	2257 (50.91)	572 (25.30)	7.832	0.020
	6–10	1877 (42.34)	438 (23.30)		
	≥ 11	299 (6.74)	55 (18.40)		
**Wealth Index**	Poor	1772 (39.97)	154 (8.70)	522.428	< 0.001
	Middle	855 (19.29)	170 (19.90)		
	Rich	1806 (40.74)	741 (41.00)		
**Birth Orders**	1^st^	1797 (40.54)	556 (30.90)	124.386	< 0.001
	2^nd^	1322 (29.82)	329 (24.90)		
	3^rd^ or above	1314 (29.64)	180 (13.70)		
**Age at First Child’s Birth (years)**	< 20	3223 (72.70)	603 (18.70)	182.75	< 0.001
	≥ 20	1210 (27.30)	462 (38.20)		
**Number of Antenatal Care (ANC) visit**	No	953 (21.50)	36 (3.80)	427.44	< 0.001
	1–2	1453 (32.78)	275 (18.90)		
	≥ 3	2027 (45.73)	754 (37.20)		
**Body Mass Index (BMI)**	Normal (18.5 kg/m^2^ ≤ BMI < 25 kg/m^2^)	2594 (58.52)	578 (22.30)	267.7	< 0.001
	Under weight (< 18.5 kg/m^2^)	1094 (24.68)	146 (13.30)		
	Over-weight (≥ 25 kg/m^2^)	745 (16.81)	341 (45.80)		
**Child’s Size at Birth**	Larger	594 (13.40)	181 (30.50)	18.66	< 0.001
	Average	2978 (67.18)	705 (23.70)		
	Smaller	861 (19.42)	179 (20.80)		
**Age at First Marriage (years)**	≤ 18	3182 (71.78)	618 (19.40)	130.86	< 0.001
	> 18	1251 (28.22)	447 (35.70)		
**Husband’s Education (level of schooling)**	No education	1014 (22.87)	92 (9.10)	374.365	< 0.001
	Primary	1343 (30.30)	204 (15.20)		
	Secondary/Higher	2076 (46.83)	769 (37.00)		
**Type of Birth**	Single birth	4405 (99.37)	1055 (24.00)	2.11	0.146
	Multiple births	28 (0.63)	10 (35.70)		
**Gender of Child**	Male	2293 (51.73)	580 (25.30)	4.2	0.040
	Female	2140 (48.27)	485 (22.70)		
**Mother’s Working Status**	No	3351 (75.59)	807 (24.10)	0.025	0.874
	Yes	1082 (24.41)	258 (23.80)		
**Husband’s Occupation**	Service/Skilled and Unskilled manual	2045 (46.13)	429 (21.00)	310.161	< 0.001
	Professional/Technical/Managerial	410 (9.25)	220 (53.70)		
	Agricultural work	1057 (23.84)	131 (12.40)		
	None/other	921 (20.78)	285 (30.90)		
**Delivery in private Sector/Hospital**	No	3409 (76.90)	249 (7.30)	2260.37	< 0.001
	Yes	1024 (23.10)	816 (79.70)		
**Exposed to media**	Yes	2744 (61.9)	883 (32.2)		< 0.001
	No	1689 (38.1)	182 (10.8)		
**Wanted indexed child**	Yes	3974 (89.6)	1000 (25.2)		< 0.001
	No	459 (10.4)	65 (14.2)		

Moreover, the wealth index was significantly associated with mode of delivery (*p* < 0.001), where the rich mothers showed the highest rate of CS delivery (41%), followed by middle (19.90%) and poor mothers (8.70%). Interestingly, the order of childbirth had an opposite relation with the prevalence of CS delivery; the birth order was ascending, while the proportion of CS cases was significantly (*p <* 0.001) declining. Mothers’ aged ≥ 20 years at their first childbirth were more inclined to CS delivery (38.2%) and the factor had a significant association (*p* < 0.05) with mode of delivery. Besides, the mothers who were greater than 18 years old during their first marriage exhibited 16.3% higher prevalence of CS than their counterparts with ≤ 18 years (35.7% vs. 19.4%; *p* < 0.001). The number of antenatal care (ANC) visits during pregnancy has significantly influenced the mode of delivery (*p* < 0.001), where the CS rate was the highest for the mothers having three or more ANC visits (37.20%). Obese mothers (45.8%) held a higher rate of CS, and BMI was significantly associated with CS delivery (*p* < 0.001). Also, child’s size at birth was significantly associated with CS (*p* < 0.001). Surprisingly, there was no association between mothers’ working status and mode of delivery. Contrary, the husband’s occupation was significantly associated with mode of delivery (*p* <0.001), and the women whose husbands worked as professional, technical, and managerial workers had a higher CS rate (53.7%). Furthermore, the prevalence of CS was significantly higher during male childbirth than female childbirth (25.30% vs. 22.70%; *p* < 0.05). Apart from, mothers exposed to media were more prone to CS delivery than mothers who were not exposed to media (32.2% vs. 10.8%; *p* < 0.001). The mothers who wanted the indexed child possessed a higher prevalence of CS delivery than their counterparts (25.2% vs. 14.2%; *p* < 0.001). Finally, mothers who got delivered in private facilities had a significantly higher CS rate than the mother who had no private facilities during childbirth (79.7% vs. 7.30%; *p* < 0.001).

### Reasons and timing of decision by decision makers

Table 2 displayed the reasons for choosing CS by decision-makers. In most cases, doctors made the final decision for CS delivery (75% cases), and family members influenced 19.25% cases, and the remaining were mothers’ decisions. Several reasons were responsible for taking CS delivery decision. Malpresentation of the fetus (32.68%) was the main factor for choosing CS delivery, followed by other complications (31.55%), failure to progress in labor (18.50%), previous caesarian section (16.53%), and so on. Convenience (9.30%) and avoiding labor pain (7.04%) were two elective choices in upward CS delivery rate, and doctor dominated both the reasons as decision-maker. More than 50% of decisions for CS delivery occurred before the delivery date, and most of the decisions have been made by a doctor (Table 3).

**Table 2:** Reasons for choosing caesarian section for delivery by decision makers based on the information reported by mothers (N = 1065).

Reasons	Mother said Count (%)	Family Members said Count (%)	Doctor said Count (%)	Total Count (% among total CS delivery)
**Convenience**	11 (12.50)	41 (15.02)	47 (4.45)	99 (9.30)
**Avoiding labor pain**	17 (19.32)	28 (10.26)	30 (2.84)	75 (7.04)
**Malpresentation of the fetus**	7 (7.95)	42 (15.38)	299 (28.29)	348 (32.68)
**Premature baby**	2 (2.27)	3 (1.10)	19 (1.80)	24 (2.25)
**Cord prolapsed**	2 (2.27)	2 (0.73)	20 (1.89)	24 (2.25)
**Multiple births**	2 (2.27)	0 (0.00)	3 (0.28)	5 (0.47)
**Failure to progress in labor**	8 (9.09)	37 (13.55)	152 (14.38)	197 (18.50)
**Pre-eclampsia**	3 (3.41)	2 (0.73)	20 (1.89)	25 (2.35)
**Diabetes**	1 (1.14)	1 (0.37)	6 (0.57)	8 (0.75)
**Previous caesarean section**	16 (18.18)	46 (16.85)	114 (10.79)	176 (16.53)
**Less pressure on baby’s brain**	5 (5.68)	5 (1.83)	54 (5.11)	64 (6.01)
**Other complications**	14 (15.91)	65 (23.81)	257 (24.31)	336 (31.55)
**Other**	0 (0.00)	1 (0.37)	36 (3.41)	37 (3.47)
**Total (%)**	88 (6.20)	273 (19.25)	1057 (74.55)	

**Table 3:** Timing of decision for CS delivery by decision makers (N = 1065).

Timing of decision	Total (%)	Respondent said N (%)	Family member said N (%)	Doctor said N (%)
Day of Delivery	488 (45.8)	21(4.3)	92 (18.9)	375 (76.8)
A Day Before Delivery	143 (13.4)	2 (1.4)	23 (16.1)	118 (82.5)
2 to 7 Days Before Delivery	112 (10.5)	6 (5.4)	28 (25)	78 (69.6)
8 to 30 Days Before Delivery	94 (8.8)	8 (8.5)	13 (13.8)	73 (77.7)
Above 30 Days Before Delivery	228 (21.4)	28 (12.3)	58 (25.4)	142 (62.3)
**Total (%)**	**1065 (100)**	**65 (6.1)**	**214 (20.1)**	**786 (73.8)**

### Two-level logistic regression analysis

The estimated MOR value was 1.715, which indicated that the cluster effect was present in the current study. So, we considered two-level logistic regression analysis upon the cluster effect to get more accurate results. The findings of the two-level logistic regression model in terms of adjusted odds ratio (AOR), standard error (SE), *p*-values, and their associated confidence interval (CI) at 95% have been summarized in Table 4.

**Table 4:** Two-level logistic regression model to identify the factors associated with the CS delivery for the ever-married women of reproductive age in Bangladesh (N = 4433).

Factors	Adjusted Odds Ratio (AOR)	SE (Standard Error)	*p*-value	95% Confidence Interval for AOR	
**Division**				Lower	Upper
Dhaka^R^					
Chittagong/Chattogram	0.59	0.116	0.008**	0.401	0.870
Barishal	0.83	0.191	0.414	0.526	1.302
Khulna	1.14	0.235	0.537	0.757	1.704
Rajshahi	0.87	0.189	0.521	0.569	1.331
Rangpur	0.62	0.142	0.036*	0.395	0.969
Sylhet	0.51	0.120	0.004**	0.318	0.806
**Mothers Age (years)**					
≤ 24 ^R^					
25–35	1.73	0.277	0.001**	1.265	2.371
≥ 36	1.63	0.553	0.150	0.838	3.170
**Place of Resident**					
Urban^R^					
Rural	0.66	0.090	0.002**	0.502	0.858
**Maternal Education (level of schooling)**					
No education^R^					
Primary	0.99	0.241	0.968	0.614	1.597
Secondary	1.19	0.290	0.479	0.737	1.918
Higher	1.45	0.429	0.209	0.812	2.589
**Religion**					
Muslim^R^					
Non-Muslim	1.28	0.266	0.228	0.855	1.928
**Family Member**					
≤ 5^R^					
6–10	0.90	0.105	0.344	0.712	1.126
Above 11	0.48	0.119	0.003**	0.295	0.781
**Wealth Index**					
Poor^R^					
Middle	1.61	0.281	0.006**	1.148	2.269
Rich	1.80	0.307	0.001**	1.287	2.512
**Birth Order**					
1^st R^					
2^nd^	0.70	0.105	0.018*	0.524	0.942
3^rd^ or above	0.50	0.103	0.001**	0.334	0.747
**Age at First Birth (years)**					
< 20^R^					
≥ 20	1.12	0.179	0.484	0.818	1.530
**Number of Antenatal Care (ANC) received**					
No^R^					
1–2	2.31	0.535	< 0.001**	1.469	3.640
≥ 3	3.68	0.840	< 0.001**	2.355	5.760
**Body Mass Index (BMI)**					
Normal^R^ (18.5 kg/m^2^ ≤ BMI <25 kg/m^2^)					
Under weight (< 18.5 kg/m^2^)	0.90	0.133	0.461	0.670	1.199
Over-weight (≥ 25 kg/m^2^)	1.44	0.204	0.011*	1.088	1.897
**Child Size at Birth**					
Larger^R^					
Average	0.61	0.095	0.001**	0.449	0.825
Smaller	0.76	0.146	0.159	0.526	1.111
**Age at First Marriage**					
≤ 18^R^					
> 18	1.05	0.162	0.761	0.774	1.419
**Husband’s Education (levels of schooling)**					
No education^R^					
Primary	0.96	0.182	0.819	0.660	1.389
Secondary/Higher	1.13	0.223	0.534	0.768	1.664
**Type of Birth**					
Single^R^					
Multiple	3.87	2.390	0.029*	1.153	12.986
**Gender of Child**					
Male^R^					
Female	0.88	0.097	0.235	0.705	1.090
**Mothers Working Status**					
No^R^					
Yes	1.07	0.139	0.605	0.829	1.381
**Husband’s Occupation**					
Service/Skilled and Unskilled manual^R^					
Professional/Technical/Managerial	1.68	0.330	0.008**	1.147	2.473
Agricultural work	1.18	0.196	0.326	0.850	1.630
None/other	1.20	0.171	0.203	0.907	1.585
**Delivery in private Sector/Hospital**					
No^R^					
Yes	38.70	5.303	< 0.001**	29.587	50.626
**Exposed to media**					
No^R^					
Yes	0.832	0.128	0.237	0.614	1.128
**Wanted indexed child**					
No^R^					
Yes	1.07	.262	0.767	0.667	1.735

Here, R = reference group.

According to the geographical region, mothers from Chittagong/Chattogram, Rangpur, and Sylhet divisions have showed significantly lower CS rate ([AOR = 0.59, 95% CI = 0.401, 0.870; *p* = 0.008], [AOR = 0.62, 95% CI = 0.395, 0.969; *p =* 0.036], [AOR = 0.51, 95% CI = 0.318, 0.806; *p* = 0.004], respectively) than the Dhaka division. Similarly, rural mothers were less likely to have CS delivery than the urban mothers (AOR = 0.66, 95% CI = 0.502, 0.858; *p =* 0.002). Mothers aged 25–35 years were more likely to have significantly higher CS delivery than the mothers from ≤ 24 years age group (AOR = 1.73, 95% CI = 1.265, 2.371; *p =* 0.001).

Regression analysis also depicted a clear view of a significant correlation between the number of family members and prevalence of CS delivery mode, where the mothers from families containing above 11 members exhibited a lower rate of CS childbirth compared to the mothers from families with less than or equal five members (AOR = 0.48, 95% CI = 0.295, 0.781; *p* = 0.003). Besides, mothers who were economically rich and middle level had significantly higher rate of CS delivery than economically poor mothers [(AOR = 1.80, 95% CI = 1.287, 2.512; *p* = 0.001) and (AOR = 1.61, 95% CI = 1.148, 2.269; *p =* 0.006), respectively]. In contrast, CS delivery was significantly lower for second and successive childbirth than the first delivery [(AOR = 0.70, 95% CI = 0.524, 0.942; *p =* 0.018), and (AOR = 0.50, 95% CI = 0.334, 0.747; *p =* 0.001), respectively].

Moreover, mothers who had higher ANC visits were more inclined to CS delivery; for instance, mothers who had 1–2 and ≥ 3 ANC visits displayed 2.3 times (AOR = 2.31, CI = 1.469, 3.640; *p <* 0.001), and 3.68 times (AOR = 3.68, 95% CI = 2.355, 5.760; *p* < 0.001) higher CS delivery, respectively, than the mothers who had no ANC visit. Notably, the chance of CS delivery was more than 38 times higher if the delivery occurred in the private hospital (AOR = 38.7, 95% CI = 29.587, 50.626; *p <* 0.001). Furthermore, the obese mothers (BMI ≥ 25 kg/m^2^) showed 1.4 times higher CS rate (AOR = 1.44, 95% CI = 1.088, 1.897; *p =* 0.011) than the mothers who were in normal range of BMI (18.5 kg/m^2^ ≤ BMI < 25 kg/m^2^). Finally, the mothers who delivered average size babies had fewer chance of CS (AOR = 0.61, CI = 0.449, 0.825; *p =* 0.001) than the mothers who delivered larger size babies.

However, the regression analysis has traced no significant variation of CS rate among the mothers from various educational levels from the reference group (mothers with no education) (*p* > 0.05). Similarly, mothers’ religious status, age at first childbirth, age at first marriage, working status, exposure to media, including wanted indexed child and husband’s education level, resulted in no significant CS rate difference among various groups from their corresponding reference groups.

## Discussion

This study aimed to identify socio-economic, demographic, institutional, and social-network-related risk factors associated with CS delivery based on data obtained from a nationally representative survey. The prevalence of CS delivery was found at 24%, which was substantially higher than the ideal value recommended by WHO (10% to 15%) [3]. The current finding is too alarming because the CS delivery rate of Bangladesh was only 2.37% in 2000 [26]. It is a common trend that the CS delivery rate considerably varies in different regions of the world concerning differences in socio-economic conditions, culture, and education. According to a global survey by WHO, CS delivery was the highest in China (46.2%) and lowest in Cambodia (14.7%) in Asia [27]. Besides, this method of delivery rate was the highest in Brazil (45.9%) and lowest in Chad (0.4%), and in our two neighboring countries (India and Pakistan) was around 7% [28].

The current study revealed that 9.3% and 7% of CS delivery occurred for convenience and avoiding labor pain, respectively, and mothers and their family members played a dominating role in these two reasons as decision-makers. The present results also stated that about 74% of mothers decided CS delivery as doctors or physicians had suggested, and most of the time, physicians determined to CS childbirth due to complications in pregnancies, such as malpresentation of fetus (28.29%), failure to progress in labor (14.38%), and other complications (24.31%). Timing of decision showed that 40.7% of decisions of CS delivery had taken two or more days before the delivery date, which indicated a significant proportion of decisions of CS had taken way before the complications of delivery had arisen.

Bangladesh has been getting tremendous success in the improvement of per capita income for the last couple of decades. This study demonstrated that the wealth index was significantly associated with increased in CS delivery which is alarming. Mothers from higher-income family usually live with more comforts and facilities, and therefore, might be more anxious about the labor pain of vaginal delivery and might go under CS delivery. This outcome might be regarded as consistent and replicable to the previous findings [8, 9, 26]. A study explicated that the CS delivery rate was increasing with the upward of average income in both urban and rural areas in Bangladesh, and the rate was higher in the urban area compared to the rural area [26]. Similarly, the current analysis demonstrated a significantly higher CS rate in urban mothers compared to the rural mothers of Bangladesh. In the country, most of the medical facilities are available in the city area. The mothers of the urban site can easily access the required nursing care from various private or public care during pregnancy. Besides, above 74% of city dweller women are educated and belong to wealthy or middle-class families capable of undergoing CS mode delivery [8].

In Bangladesh, the ANC rate has increased in the last two decades [29], and mothers who take ANC from private network are more prone to CS delivery [30]. The current analysis proved that mothers having one or more ANC visits were more likely to have CS delivery than the mothers who had no ANC visit. Delivery in private facilities was found as a prominent influencer to increase the risk of CS delivery rate. In the last two decades, the private sector in Bangladesh has flourished dramatically. The private sector is very much profit generative, and physicians in private hospitals get incentives for conducting surgery, and that may influence some physicians to suggest mothers go under CS delivery. There might be some other reasons that increase CS delivery rate in private facilities, such as the perception of providing improved care and availability of modern technologies and regular specialist physicians. On the other hand, lower trust level on public hospitals’ provided care, lack of enough specialist doctors and nurses, and insufficient health services and medicines, and so on, are the main reasons for keeping away from public hospital during the delivery time [31, 32]. In addition, a fixed obstetrician always investigates a pregnant mother that might be another notable reason for choosing CS delivery in private hospitals.

The birth order of the newborn was also significantly associated with CS delivery. The babies with 2^nd^ or higher birth orders were less likely to be delivered by CS method than the babies with 1st birth order. The possible explanation is that mothers are more fearful about the labor pain and bleeding at their first delivery. Besides, complications in first delivery are higher than in the second and successive deliveries [33], and that might intimidate mothers to choose CS delivery. Another explanation would be after having CS delivery in the first birth; subsequent deliveries are perceived as high risk, thus decreasing the likelihood. This study also revealed that the geographical region was also significantly associated with CS delivery. Mothers who live in Dhaka and Urban areas were more prone to CS delivery. The availability of private facilities and the higher number of ANC visits contribute to these findings.

Early and delayed pregnancy in the reproductive age might be considered at risk for adverse pregnancy outcomes. Several studies reported an association of advanced maternal age with preterm delivery, low birth weight, perinatal death, and CS [34, 35]. Similarly, this research found an association between mothers’ age and CS delivery. Mothers aged greater than 25 years were more likely to CS delivery compared to their young counterparts. With the increase in education rate, expansion of the social network, and exposure to cultures of different countries, mothers in Bangladesh are embarking on having a child at a later age. As a result, the chance of CS delivery was higher for them. Furthermore, mothers from families with higher members were less likely to have CS delivery. One reason may be that family bonding among the members of large family is strong, and mothers get motivation and psychological strength from the other members of the family for vaginal delivery. The economic condition might be another contributing factor in this circumstance.

Obesity was significantly associated with a higher rate of CS delivery. Pieces of evidence showed that overweight mothers are at risk of gestational diabetes, induction of labor, miscarriage, preeclampsia, venous thromboembolism, anesthetic complications, and wound infections [36]. As a result, mothers could choose CS delivery mode. This study also showed that child size at birth, twin birth, and occupation of husband were associated with an increasing rate of CS delivery. Although the current regression analysis revealed no significant differences between CS delivery and mother’s education, gender of the child, and exposure to media, significant associations were found from bivariate analysis. It has been noticed that with the increase in mothers’ education level, CS delivery increase noticeably. Another study with different settings has found mothers’ education significantly related to CS delivery [8]. We have also observed that the prevalence of CS delivery was more remarkable for the male than the female, which indicated gender inequality. Sometimes CS is justifiable but should not be occurred because of being a female child. Another alarming finding was that the prevalence of CS delivery was greater for the mothers who were exposed to the media than the mothers who were not exposed.

Several more factors associated with CS delivery in Bangladesh are still to be determined. However, some effective measures considering factors that we have identified in this study might extensively reduce the CS delivery rate.

Since the analysis of study was based on retrospective data, the obtained results might be subjected to be potentially biased. Some significantly associated factors found in several other studies were not available in BDHS- 2014 data. Another limitation is pieces of information were collected only from the response of mothers. The collection of information from physicians would help us to do a deeper analysis. As the data was collected from a cross-sectional study, we could not observe a cause-effect relationship. However, one of the strengths of this study was that we had used nationally representative survey data.

## Conclusion

The current investigation based on BDHS-2014 data has revealed that the prevalence of CS delivery in Bangladesh was higher than that has been recommended by WHO. This study found mothers from urban areas, Dhaka division, wealthy families, and had a delivery in private facilities were more prone to CS delivery. Mothers’ age and education, childbirth order, number of ANC visits, larger baby size at birth, twin childbirth, and husbands’ occupation were significant predictors of CS delivery. So, it is high time to take necessary steps considering these factors to control the growing trend of CS delivery rate. It is highly recommended that clinics should be audited regularly, and there must have some strict instructions and proper guidelines when conducting CS is justifiable. Besides, some intervention programs could be carried out to raise public awareness against the harmful consequences of unnecessary CS delivery.
